# Ultrafast Hot Carrier Cooling Enabled van der Waals Photodetectors at Telecom Wavelengths

**DOI:** 10.1021/acs.nanolett.4c05953

**Published:** 2025-02-24

**Authors:** Zhouxiaosong Zeng, Yufan Wang, Patrick Michel, Fabian Strauß, Xiao Wang, Kai Braun, Marcus Scheele

**Affiliations:** † Institute of Physical and Theoretical Chemistry and LISA+, University of Tübingen, Auf der Morgenstelle 18, D-72076, Tübingen, Germany; ‡ School of Physics and Electronics, Hunan University, Changsha 410082, China; § Key Laboratory for Micro-Nano Physics and Technology of Hunan Province, College of Materials Science and Engineering, Hunan University, Changsha 410082, China

**Keywords:** Graphene, Photodetectors, Hot Carriers, Ultrafast Photocurrent, Photothermionic Effect, Telecom Wavelength, Pump−Probe

## Abstract

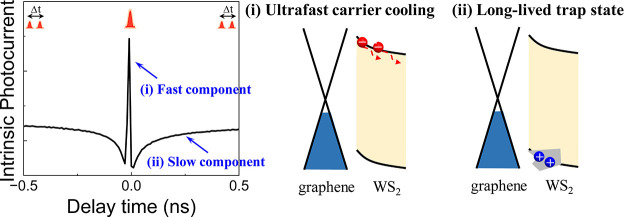

Two-dimensional (2D) materials with high carrier mobility and picosecond intrinsic response times exhibit large potential for fast optoelectronics operating at telecom wavelengths. However, due to the inefficient utilization of hot carriers, 2D photodetectors for the telecom C-band have rarely been realized. Here, we report a high-performance and waveguide-free WS_2_/graphene photodetector operating at 1560 nm enabled by hot carrier injection and long-lived charge separation. The efficiently injected hot electrons from graphene to WS_2_ exhibit ultrafast cooling dynamics with a 3 ps intrinsic response time. Simultaneous hole trapping in WS_2_ ensures a long circulation of injected electrons, enabling a high responsivity of 0.26 A/W. In continuation, we present a vertical WS_2_/graphene/WSe_2_ device with a built-in electric field and the same detection mechanism, for which a photocurrent on–off ratio of 3500 and an extrinsic response time of 1.71 ns are obtained. Our study highlights the benefit of combining 2D semiconductors and semimetals for high-speed photodetection.

Fast photodetectors that convert an on-chip optical signal into an electrical output at high-speed play vital roles in modern imaging, sensing, and communication systems.^1^ Operation at telecom wavelengths is especially preferred due to low transmission losses and the availability of optical gain. Two-dimensional (2D) transition metal dichalcogenides (TMDCs) with bond-free van der Waals (vdW) structures possess relatively large carrier mobilities^2−4^ and intrinsic response times on the order of picoseconds,^5−7^ demonstrating great potential for the next generation of ultrafast photodetectors.

One of the most important challenges for the development of information technology is the large heat dissipation caused by long-distance data transmission and fast signal conversion in high densities.^8^ A major source for such electrical heat is the generation of hot electrons, which refers to high-temperature carriers beyond the thermal equilibrium with the lattice.^9^ If this electrical heat could be effectively harnessed, heat dissipation would be reduced and the power conversion efficiency of the device simultaneously enhanced.^10^ A promising strategy in this respect is the thermionic emission of photoexcited electrons. Briefly, if the photon energy of the optical signal is higher than the Schottky barrier between a metal and a semiconductor, the photogenerated hot electrons in the metal can be extracted and injected into the adjacent semiconductor after their thermalization.^11^ This photoelectric effect enables a sub-bandgap photoresponse at telecom wavelengths in 2D TMDCs without sacrificing response speed. However, due to the relatively weak light absorption by the metal, the conventional metal-semiconductor-metal structure requires an integration into silicon waveguides to enhance the hot electron injection efficiency,^12,13^ which makes the device configuration sophisticated and limits the response to certain wavelengths.

In a related strategy, graphene has been reported as a material for the electrical thermal management of nanoscale devices due to its high thermal conductivity,^14^ low heat capacity,^15^ and unique hot carrier cooling dynamics.^16,17^ After excitation, the photogenerated charge carriers in graphene can thermalize themselves via carrier scattering with a temperature of hundreds of Kelvins higher than the lattice, inducing excess electrical heat.^16^ With its gapless band structure and vdW property, combining graphene and TMDCs to construct heterostructure devices targets the utilization of these hot electrons for photodetection at telecom wavelengths with highly efficient hot carrier injection, reduced wasted heat, and low dark currents. The charge carrier dynamics in graphene/TMDC heterostructures have been extensively investigated via all-optical pump–probe methods, where hot carrier transfer or injection from graphene to adjacent layers over the Schottky barrier is reported at the picosecond time scale.^18,19^ Further terahertz and all-optical pump–probe studies showed that the injected hot electrons can be trapped by the defects^20^ in the TMDC or recycled via electron–phonon interactions^21^ from the heterostructure. This way, the injected carrier lifetime could be extended to nanoseconds, which enabled the harvesting of hot carriers over realistic device lengths. This is an important prerequisite for solving the trade-off between high response speed and high responsivity in such a device. However, a demonstration of this principle in actual devices via optical pump-photocurrent probe experiments have not been reported so far.

Here, we construct a waveguide-free WS_2_/graphene photodetector and apply the two-pulse coincidence (2PC) photoresponse technique via asynchronous optical sampling (ASOPS) to determine the hot carrier dynamics that contributes to the photocurrent. Upon 1560 nm excitation, the heterostructure photodetector exhibits two lifetime components with τ_fast_ ≈ 3 ps and τ_slow_ ≈ 500 ps. We attribute the τ_fast_ to a hot carrier injection and cooling, which we rationalize in terms of its superlinear power-dependence, the positive contribution to the photocurrent, and an increasing lifetime with decreasing temperature. We argue that the slow component resembles trapped holes in the TMDC, which is supported by its negative contribution to photocurrent and its disappearance upon applying large biases. The fast hot carrier cooling and long circulation of electrons in the heterostructure afford a telecom C-band-suited photodetector with a simultaneously enhanced photoresponse speed and responsivity. Moreover, we show that the photodetection performance can be further increased by designing a vertical WS_2_/graphene/WSe_2_ device with an out-of-plane built-in electric field and the same hot carrier injection mechanism. For this device, the photocurrent on–off ratio is 3500 with an extrinsic response time of 1.71 ns and a responsivity of 0.18 A/W.

The WS_2_/graphene heterostructure in this study was fabricated via a standard all-dry transfer technique and low-temperature annealing. E-beam lithography (EBL) and thermal evaporation were then employed to define Au/Cr electrodes. Before transferring a WS_2_ layer on an exfoliated graphene flake, oxygen plasma treatment was first introduced to etch the graphene into a desired shape. Figure 1a exhibits an optical microscopy image of a typical device, where both electrodes were fabricated on top of thin-layered WS_2_. A Raman spectrum (Figure 1b) collected at the heterostructure region indicates the fingerprint of WS_2_ (*2LA*(*M*) peak at 351.6)^22^ and graphene (*G* peak at 1582 cm^–1^ and *2D* peak at 2700 cm^–1^).^23^

**Figure 1:**
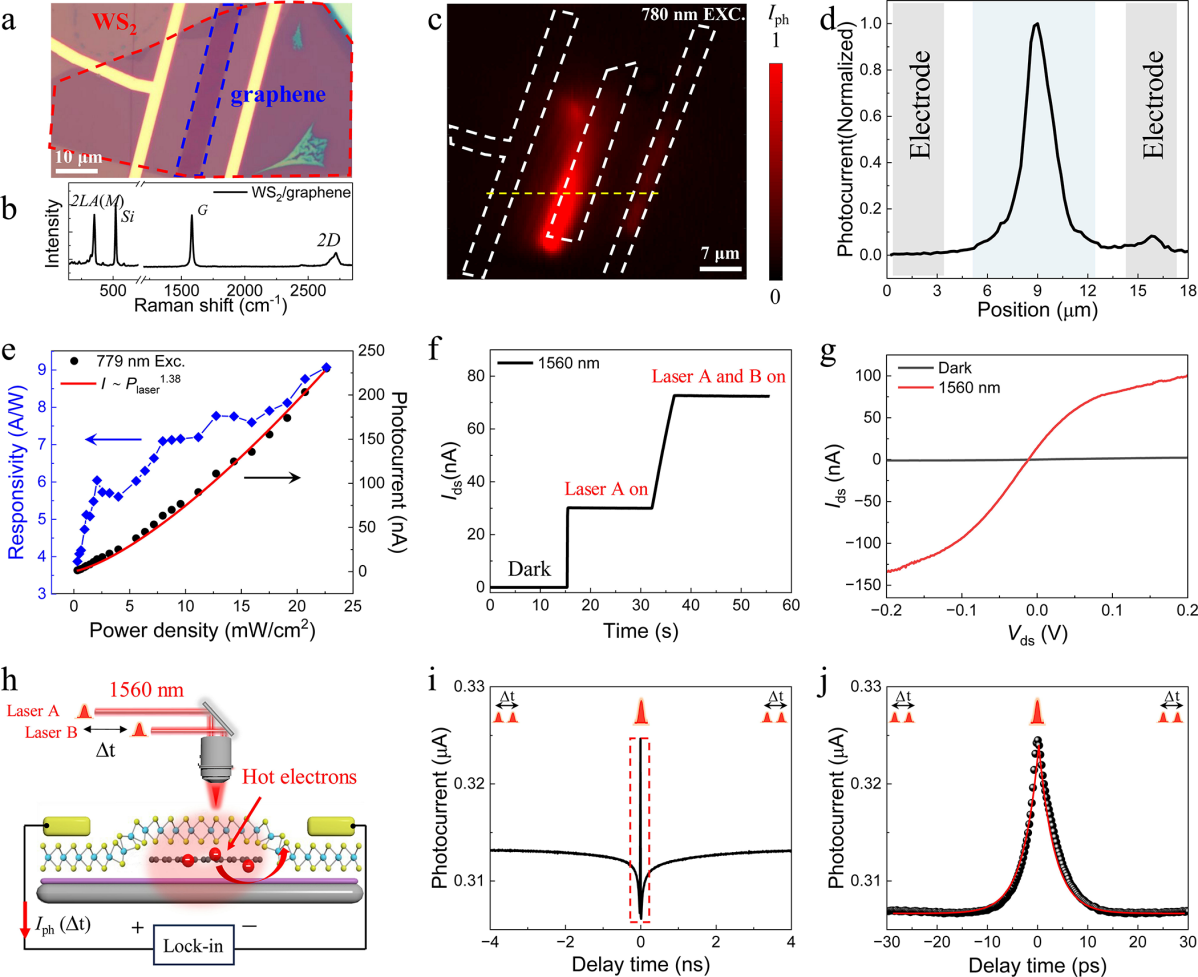
Hot carrier injection enabled WS_2_/graphene photodetector. (a) Optical image of a WS_2_/graphene photodetector, where the red and blue dashed lines highlight the WS_2_ and graphene layers, respectively. (b) Raman spectrum of the heterostructure. (c) Scanning photocurrent microscopy showing the spatially resolved photoresponse under sub-WS_2_ bandgap excitation, where the dashed white lines indicate the positions of electrodes and heterostructure overlayer. The color bar represents the normalized photocurrent intensity. (d) The extracted photocurrent line profile along the dashed yellow line in c. The light blue area indicates the position of the heterostructure. (e) Excitation power density dependence of responsivity (left) and photocurrent (right) under a 779 nm (sub-WS_2_ bandgap) pulsed laser excitation. The red line is a power-law fit with *I*_pc_ ≈ *P*^1.38^. (f) Time-resolved photoresponse under 1560 nm excitation. Both laser A and laser B have a similar output power of approximately 90 mW. (g) *I*–*V* characteristic curves in the dark (black) and under 1560 nm illumination (red). The small short-circuit current could originate from the unbalanced illumination from a laser fiber. (h) Schematic illustration of two-pulse coincidence (2PC) measurement with 1560 nm excitation. (i) 2PC measurement under a bias of 0.3 V, where the change in photocurrent Δ*P* induced by the probe laser is plotted as a function of delay time Δ*t*. (j) Zoomed-in 2PC measurement result corresponding to the red rectangle region in i.

We investigate the photoresponse in the heterostructure device by a scanning photocurrent microscope (SPCM). Under sub-WS_2_ bandgap (780 nm) excitation, we find a photocurrent with unchanged polarity at the heterostructure region and detect only a weak signal at the electrode edges (Figure 1c), presumably originating from the hot carriers generated in graphene. This is detailed further in the extracted photocurrent line profile (Figure 1d), where the normalized photocurrent intensity at the heterostructure center is 1 order of magnitude higher than that at the electrodes. Upon increasing the excitation power (Figure 1e, sub-WS_2_ bandgap excitation), the obtained photocurrent demonstrates a unique superlinear dependence with a fitted power law of *P* ∼ 1.38 (black dots and red line). This is in contrast to the conventional saturated absorption with sublinear power dependence (Supporting Information Section 1), and we interpret this finding with thermal activation of carriers over the Schottky barrier between WS_2_ and graphene.^11^ In addition, the device exhibits a high responsivity of approximately 9 A/W at a 779 nm and 22.6 mW/cm^2^ excitation power density (blue squares).

We now investigate the device behavior under 1560 nm excitation. We use two lasers A and B (see methods: ASOPS) with a similar output power of 90 mW. We find a net photocurrent of 30 nA induced by only laser A (the first step in Figure 1f) which increases by 40 nA upon adding the illumination by laser B (the second step in Figure 1f). This result confirms the superlinear behavior also at telecom wavelengths, indicative of hot carrier injection. The I–V characteristics (Figure 1g) displays a pronounced photocurrent under illumination by both lasers and a dark current roughly 8 orders of magnitude smaller than in pure graphene (Supporting Information Section 3).

We further investigate the hot carrier dynamics by two-pulse coincidence (2PC) measurements in vacuum using ASOPS. Briefly, the device is excited by the two pulsed lasers A and B (pump and probe) separated by a delay time Δ*t*. The corresponding photoresponse collected by the lock-in amplifier is recorded as a function of delay time Δ*t* between the pulses (Figure 1h) and hence the technique is also called photocurrent autocorrelation.^24^ In a typical 2PC measurement, when the pump and the probe lasers coincide spatially and temporally, the generation of photocurrent by the probe is suppressed because of the saturation in the ground state, causing a prominent dip at the zero time delay Δ*t* = 0. With an increase in the delay time, parts of the pump-induced charge carriers relax, and they can be excited again by the probe beam. Hence, one would expect an exponential recovery of photocurrent with the delay time Δ*t*. Our WS_2_/graphene heterostructure device exhibits two lifetime components (Figure 1i) with τ_fast_ ≈ 3 ps (Figure 1j) and τ_slow_ ≈ 500 ps (Supporting Information Section 4), indicating the presence of two distinct dynamic processes. The fast component appears as a peak (positive contribution to the photocurrent) and the slow component as a dip (negative contribution).

To understand the provenance of these two components, we first analyze the fast component. By studying its dependence on the external bias and temperature, we test our previous hypothesis from the 2PC experiment that this component originates from cooling of injected hot carriers (Figure 2b and 2c). Varying the external voltage from 0.5 to 3.5 V has no significant effect on the response time, which argues against transport-related mechanisms,^25^ such as charge carrier drift. The negative correlation with temperature, e.g. the observed increase of the lifetime from 3 to 5.2 ps while decreasing the temperature from 300 to 80 K, rules out a recombination-related process.^7^ We argue that this phenomenon is best explained with phonon-mediated cooling, where additional energy is needed to activate phonons at low temperature, which slows the cooling.^26,27^ It should be noted that due to the energy of the input laser (0.795 eV) is smaller than half of the bandgap energy of thin layered WS_2_ (∼0.96 eV), the two-photon absorption is not responsible for the fast component. The results also indicate that the ultrafast hot carrier cooling in pure graphene is preserved in the WS_2_/graphene heterostructure, leading to an intrinsic response time comparable to pure graphene.^17^

**Figure 2:**
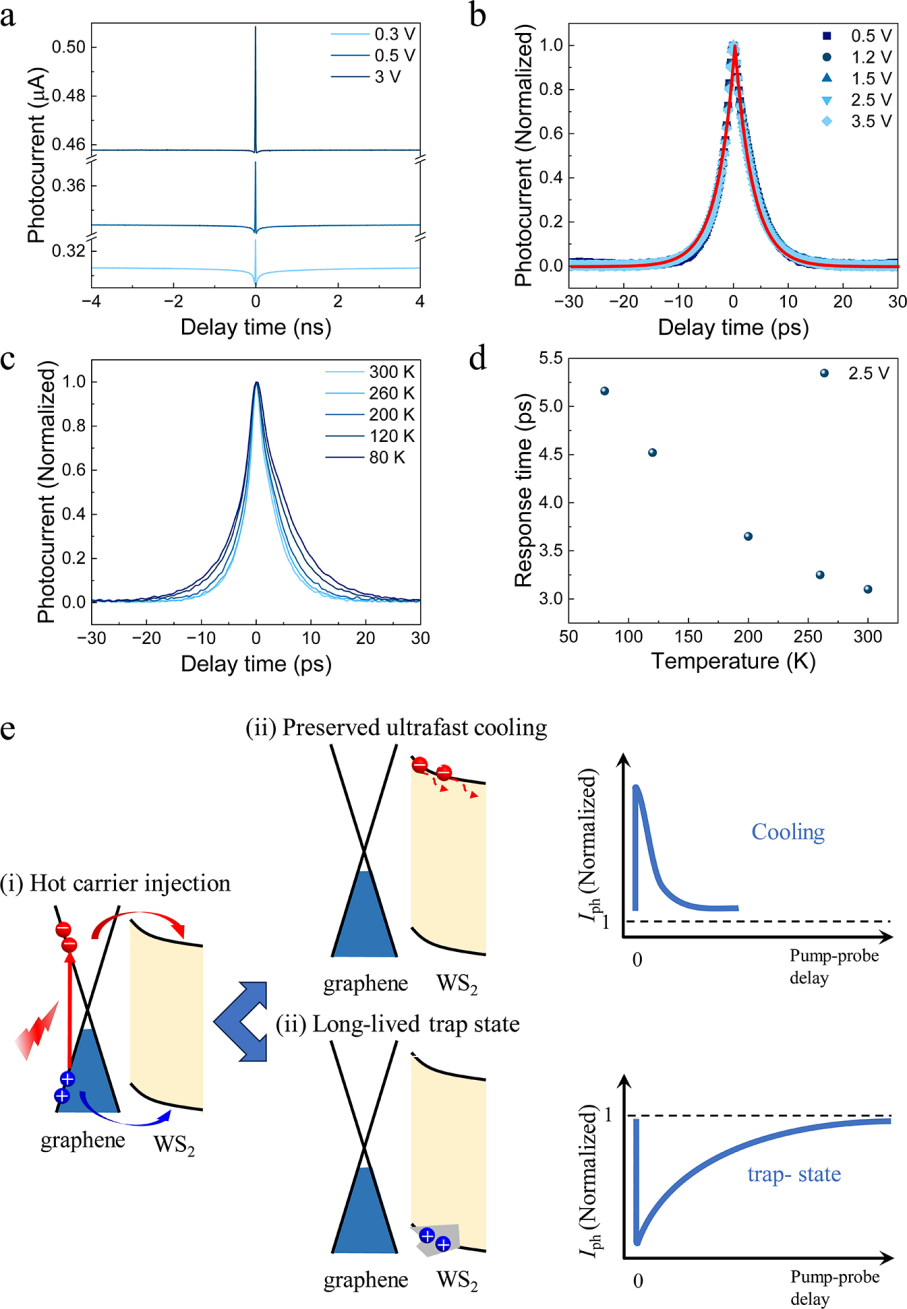
Two-pulse coincidence (2PC) measurements of the WS_2_/graphene photodetector. (a) External voltage dependence of 2PC results. (b) External voltage dependence of the 2PC results for a time delay from −30 to 30 ps. (c) Temperature dependence of 2PC results for a time delay from −30 to 30 ps. (d) Corresponding temperature-dependent intrinsic response times. (e) Schematical illustration of hot carrier dynamics in WS_2_/graphene. Upon illumination, the generated electrons and holes in graphene are injected into WS_2_ (i). The injected hot electrons possess ultrafast cooling similar to that of pure graphene and demonstrate a fast peak signal in 2PC measurement (ii, upper plane), while the injected holes are trapped by the surface defects at the WS_2_ and contribute to a long circulation of injected electrons, which demonstrate a slow signal dip in 2PC measurement (ii, lower panel).

We continue with analyzing the slow component and argue that it is related to carrier trapping, similar to a recent report for WS_2_ based on terahertz spectroscopy.^20^ In detail, upon illumination by the 1560 nm laser, electrons and holes in graphene are rapidly injected into the adjacent WS_2_ layer (Figure 2e i). After that, a fraction of the hot electrons cool in several picoseconds, inducing an ultrafast intrinsic response peak in the 2PC signal (Figure 2e ii, upper panel). Meanwhile, the injected holes are captured by the defects at the surface of WS_2_, which not only leads to a relatively long circulation of the electrons, reflected by the slow intrinsic response dip in the 2PC signal (Figure 2e ii, lower panel), but also contributes to a high responsivity of 0.26 A/W (see Supporting Information Section 7 for detailed calculations). It should be noted that both injected electrons and holes could be trapped by the surface defects. Considering the n-type nature of thin-layered WS_2_, only the trapping of the minority carrier, that is the hole, favors the enhancement of the photocurrent and is consistent with our observations. In comparison, the device without the slow dip component possesses 1 order of magnitude lower responsivity under the same measurement condition (Supporting Information Section 8). In addition, upon increasing the external bias, the dip can be almost entirely suppressed (Figure 2a), presumably due to the depopulation of traps.

The dominant ultrafast hot carrier cooling intrinsic response in the WS_2_/graphene heterostructure indicates its high potential for high-speed photodetection. In contrast to the intrinsic response time reflecting an inherent carrier dynamics, the extrinsic response of a photodetector includes additional effects of the device geometry (e.g., its RC time) and serves as a measure for the conventional device speed. To this end, we construct a WS_2_/graphene photodetector array (Figure 3b) via oxygen plasma etching and all-dry transfer processes (Figure 3a and Supporting Information Section 9) and determine the extrinsic photoresponse using the experimental setup illustrated in Figure 3c. Upon 779 nm pulse illumination (sub-WS_2_ bandgap), the device displays a constant response for several cycles as shown in Figure 3d. We exemplarily extract a single pulse response and resolve it on a semilog scale axis. The 90% to 10% photocurrent decay indicates an extrinsic response time of 7.11 ns (Figure 3e). Using fast Fourier transformation (FFT), we calculate the electrical bandwidth under the single excitation frequency and obtain a 3 dB bandwidth of approximately 17.7 MHz (Figure 3f), which is comparable to that of state-of-the-art photodetectors^28,29^ for telecom wavelengths.

**Figure 3:**
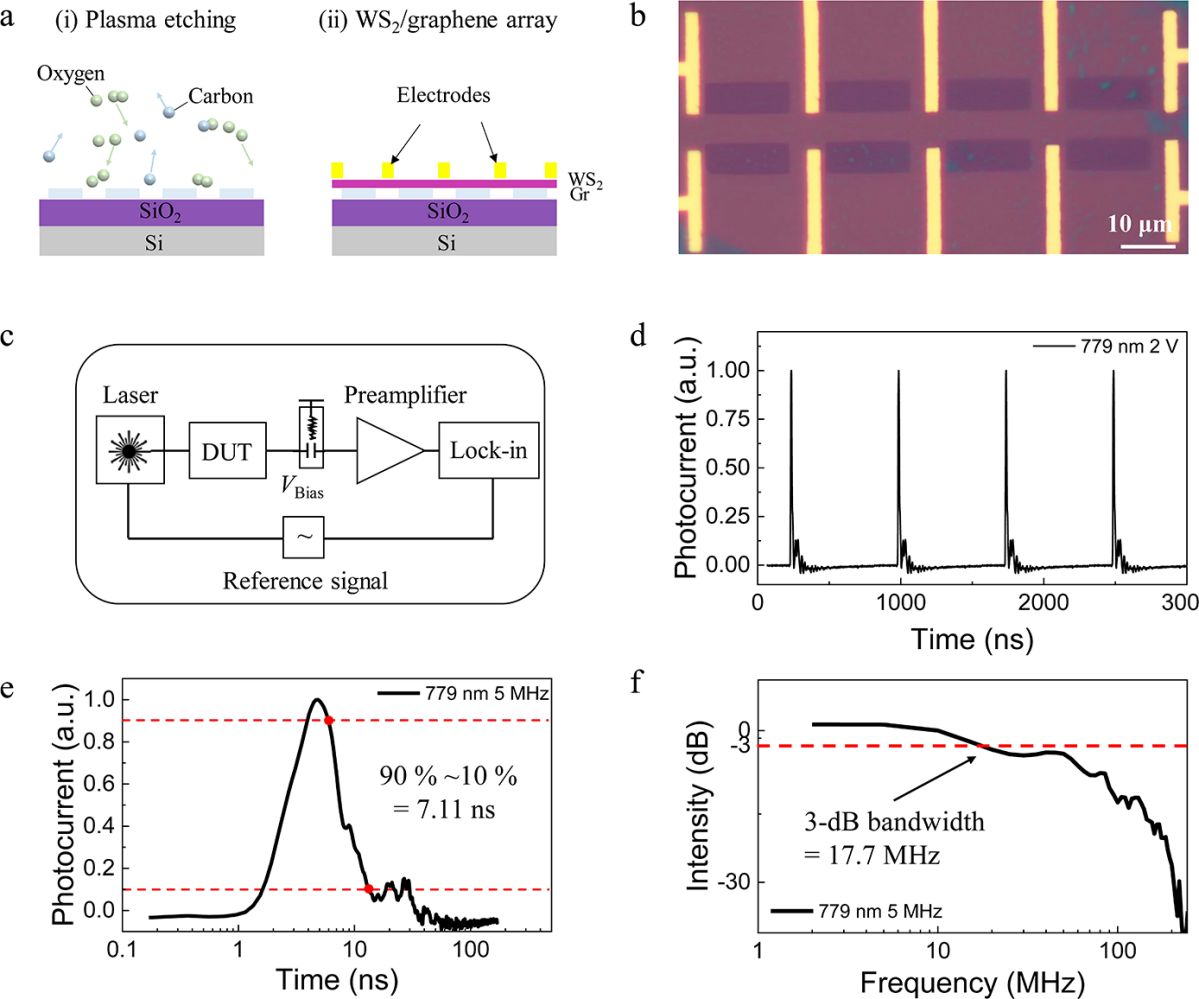
Extrinsic and 3 dB bandwidth of the WS_2_/graphene photodetector array. (a) Schematic illustration of the array fabrication processes. (b) Optical image of a WS_2_/graphene photodetector array. (c) Experimental configuration for the extrinsic response time measurements. DUT, device under test. (d) Photoresponse toward 779 nm (sub-WS_2_ excitation) pulse sequence under 2 V external voltage. (e) Normalized extrinsic response time result in semilog scale axis toward a 779 nm laser with a 5 MHz repetition frequency. The extrinsic response time defined by the 90% to 10% photocurrent decay (indicated by red dots and dashed line). (f) Fourier transformed 3 dB bandwidth corresponding to the result in e.

To further improve the performance of graphene/TMDC heterostructures based on the ultrafast hot carrier extraction and cooling, we fabricate a vertical WS_2_/graphene/WSe_2_ heterostructure (Figure 4a and 4b). In 2PC measurements (Supporting Information Section 10), the device demonstrates an intrinsic charge carrier dynamics similar to that in the WS_2_/graphene heterostructure, indicating the same photothermionic detection process. In the corresponding SPCM experiment (Figure 4c) the photocurrent appears at the heterostructure region, proving the contribution of hot carriers from the graphene. The advantage of this new heterostructure is an additional vertical built-in electric field, which is expected to reduce the out-of-plane charge carrier drift time. The I–V characteristics displays the current rectifying transport behavior (Figure 4d) and an on–off ratio of 3500, much higher than that of the lateral device. Furthermore, we obtain a laser-frequency independent extrinsic response time of approximately 1.71 ns (Figure 4e) and a 3 dB bandwidth of 123.4 MHz (Figure 4f) at 779 nm laser excitation. Thus, the vertical WS_2_/graphene/WSe_2_ heterostructure synergistically combines the properties of photodetectors with sole hot carrier injection and that of devices with built-in electric field.

**Figure 4:**
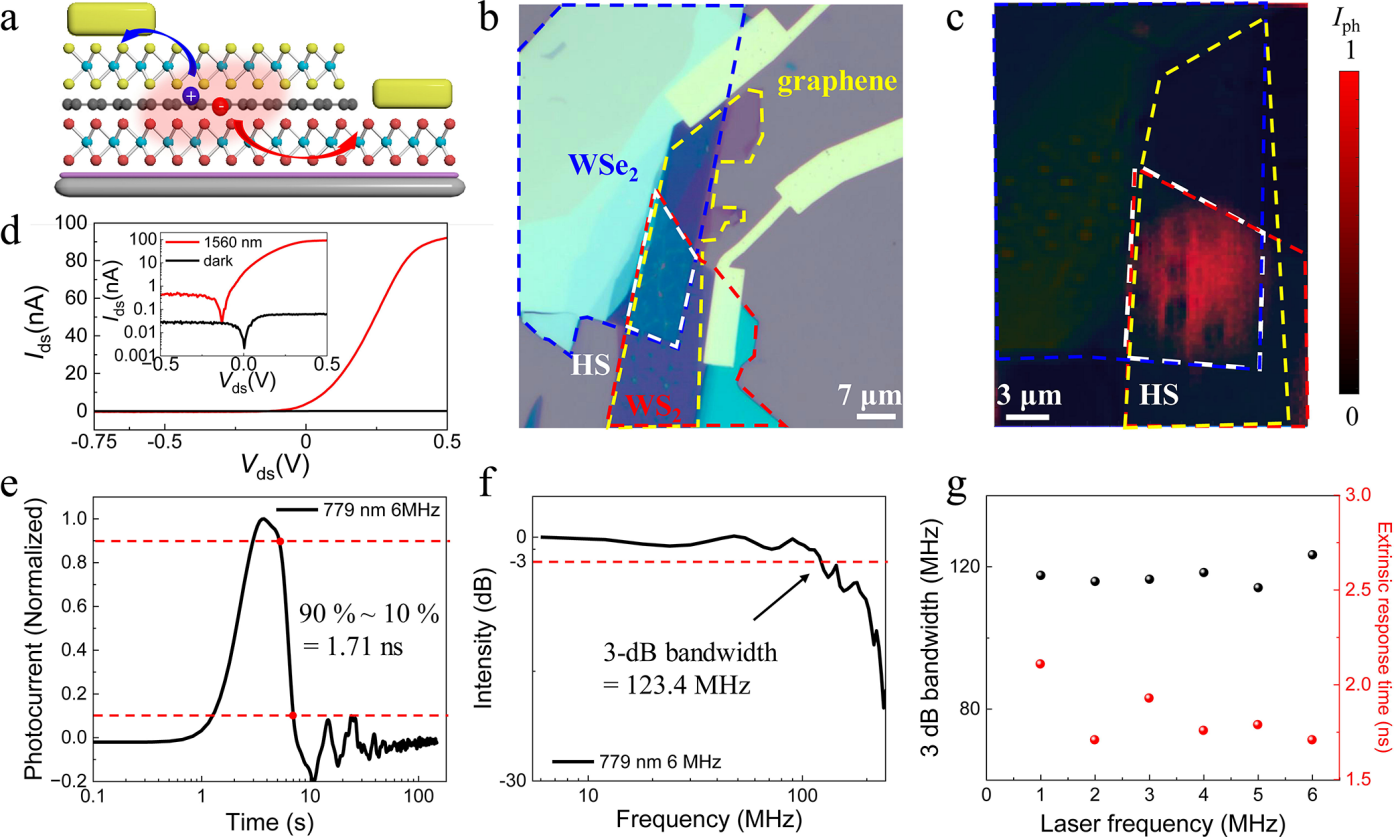
Photodetection of a WS_2_/graphene/WSe_2_ device with synergistic hot carrier injection and built-in electric field. (a) Schematical illustration of the device. (b) Optical image, where the top electrode and bottom electrode were fabricated on WS_2_ and WSe_2_ layers, respectively. (c) Superposed corresponding reflective image and SPCM result under 780 nm illumination, where the dashed blue, yellow, red, and white lines indicate the positions of WSe_2_, graphene, WS_2_, and heterostructure overlayer, respectively. The color bar represents the normalized photocurrent intensity. (d) *I*–*V* characteristic curves in dark (black) and under 1560 nm illumination. The inset shows the result on a semilog scale. (e) Normalized extrinsic response time in semilog scale axis toward a 779 nm laser with a 6 MHz repetition frequency. (f) Corresponding 3 dB bandwidth obtained by Fourier transformation. (g) 779 nm laser frequency dependence of the extrinsic response time (red) and the 3 dB bandwidth (black).

Finally, we compare the photodetection performance of our WS_2_/graphene heterostructure with other 2D vdW devices operating at telecom wavelengths. Notably, for response time (Figure 5a) and 3 dB bandwidth (Figure 5b), most results are located at the lower right-hand corner, reflecting a trade-off between responsivity and response time. In contrast, our WS_2_/graphene heterostructure with ultrafast hot carrier cooling and long electron circulation simultaneously possesses a 3 ps intrinsic response time, and 0.26 A/W responsivity while our WS_2_/graphene/WSe_2_ heterostructure possesses a 1.71 ns extrinsic response time, 123 MHz bandwidth, and 0.18 A/W responsivity, which is among the best performing detectors in this spectral range.

**Figure 5:**
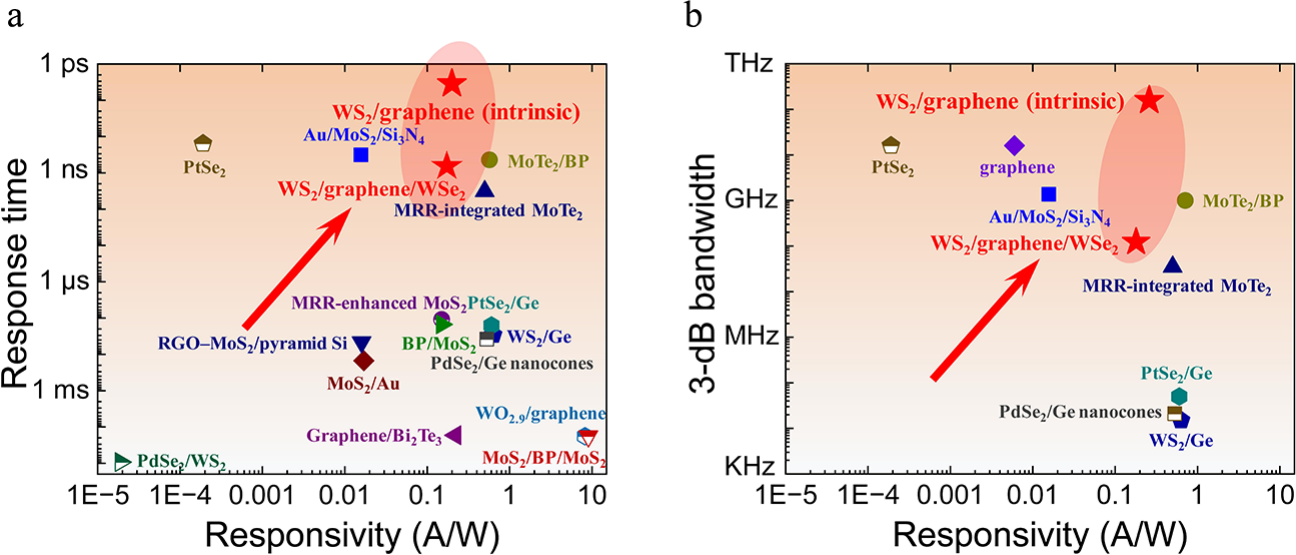
Overview of response time (a) and 3 dB bandwidth (b) and responsivity in various vdW photodetectors operating at telecom wavelengths. Data for other devices are taken from the literature (Au/MoS_2_/Si_3_N_4_, ref (12); PtSe_2_, ref (30); MoTe_2_/BP, ref (31); microring resonators (MRR)-integrated MoTe_2_, ref (32); MRR-enhanced MoS_2_, ref (13); PtSe_2_/Ge, ref (33); RGO-MoS_2_/pyramid Si, ref (34); BP/MoS_2_, ref (35); WS_2_/Ge, ref (36); PdSe_2_/Ge nanocones, ref (37); MoS_2_/Au, ref (38); WO_2.9_/graphene, ref (39); Graphene/Bi_2_Te_3_, ref (40); MoS_2_/BP/MoS_2_, ref (41); PdSe_2_/WS_2_, ref (42); graphene, ref (43)).

In summary, using the two-pulse coincidence photoresponse technique, we present graphene/WS_2_ and WS_2_/graphene/WSe_2_ photodetectors that exhibit clear signs of hot carrier injection from graphene into the van der Waals materials followed by carrier trapping. The injected hot carriers exhibit ultrafast cooling and the carrier trapping induces a long circulation of the electrons, whose combination enables our photodetector an intrinsic response time of 3 ps and a responsivity of 0.26 A/W at 1560 nm. We demonstrate sub-bandgap photodetection by these heterostructures with a 3 dB bandwidth of 123 MHz and a dark current 8 orders of magnitude smaller than in pure graphene. Our results highlight that such heterostructures synergistically combine the ultrafast response of graphene with the high sensitivity of transition metal dichalcogenides to harvest hot carriers efficiently.

## Supporting Information Available

Additional methods and experimental details, supporting sections including the power density-dependent photoresponse in WS_2_/graphene with above WS_2_ bandgap excitation, comparison of the power-dependent photocurrent in WS_2_/graphene between a 779 nm pulsed laser and a 779 nm CW laser, *I*–*V* characteristics of a pure graphene photodetector, exponential fitting of the slow dip component, power dependence of 2PC measurements in WS_2_/graphene, reproducibility of the intrinsic photoresponse signal, calculation of the photoresponsivity toward a 1560 nm laser and 779 nm impulse laser, comparison of the photocurrent intensity in devices with and without the slow component, fabrication procedure of WS_2_/graphene photodetector arrays, 2PC measurements of WSe_2_/graphene/WS_2_, supporting results for the laser repetition-related extrinsic response time and the 3 dB bandwidth in WSe_2_/graphene/WS_2_, hot carrier dynamics in WS_2_/graphene/WSe_2_ trilayer heterostructures, temperature-dependent 2PC photoresponse in a WS_2_/graphene/WSe_2_ heterostructure, as well as the extrinsic response time of WS_2_/graphene/WSe_2_ devices toward a 1310 nm square pulse laser (PDF)
