# Supplementary material for: Ultrafast Hot Carrier Cooling Enabled van der Waals Photodetectors at Telecom Wavelengths
Source: Nano Lett. 2025 Feb 24;25(9):3497–504. doi: 10.1021/acs.nanolett.4c05953 (PMC11887449; doi:10.1021/acs.nanolett.4c05953)
Supplement: Supplementary file 1 — nl4c05953_si_001.pdf [file nl4c05953_si_001.pdf]

Supporting Information

Ultrafast hot carrier cooling enabled van der Waals photodetectors at telecom wavelengths

Zhouxiaosong Zeng[1,2]†, Yufan Wang[2]†, Patrick Michel[1], Fabian Strauß[1], Xiao Wang[2,3]*, Kai Braun[1]*, Marcus Scheele[1]*

[1]Institute of Physical and Theoretical Chemistry and LISA+, University of Tübingen, Auf der Morgenstelle 18, D-72076, Tübingen, Germany

[2] School of Physics and Electronics, Hunan University, Changsha 410082, China

[3] Key Laboratory for Micro-Nano Physics and Technology of Hunan Province, College of Materials Science and Engineering, Hunan University, Changsha, 410082, China

*Corresponding authors:

E-mail addresses: xiao_wang@hnu.edu.cn(X. Wang); kai.braun@uni-tuebingen.de (K. Braun); marcus.scheele@uni-tuebingen.de (M. Scheele)

Table of content:

Methods

Supporting Section 1: Power density dependent photoresponse in WS_2/graphene heterostructures with 636 nm (above WS_2 bandgap) excitation

Supporting Section 2: Comparison of the power-dependent photocurrent generated in a WS_2/graphene heterostructure between a 779 nm pulsed laser and a 779 nm CW laser

Supporting Section 3: I-V characteristics of a pure graphene photodetector

Supporting Section 4 : Exponential fitting of the slow dip component

Supporting Section 5: Power dependence of 2PC measurements in WS_2/graphene

Supporting Section 6: Reproducibility of the intrinsic photoresponse signal

Supporting Section 7 : Calculation of the photoresponsivity for WS_2/graphene at 1560 nm laser and 779 nm impulse laser

Supporting Section 8 Comparison of the photocurrent intensity in devices with and without the slow component

Supporting Section 9: Fabrication procedure of WS_2/graphene photodetector arrays

Supporting Section 10 : 2PC measurements of WSe_2/graphene/WS_2 veritical heterostructures

Supporting Section 111 : Supporting results for the laser repetition-related extrinsic response time and the 3-dB bandwidth in WSe_2/graphene/WS_2 photodetectors

Supporting Section 12 : Hot carrier dynamics in WS_2/graphene/WSe_2 tri-layer heterostructures

Supporting Section 13 : Temperature-dependent 2PC photoresponse in a WS_2/graphene/WSe_2 heterostructure

Supporting Section 14 : Extrinsic response time of WS_2/graphene/WSe_2 devices towards 1310 nm square pulse laser

Supporting References

METHODS

Device fabrication: The WS_2/graphene devices were both fabricated based on mechanically exfoliated materials with the all-dry transfer method. Before transfer, graphene or WS_2 flakes were first mechanically exfoliated from single crystals onto transparent polydimethylsiloxane (PDMS). Thereafter, the graphene flake was placed onto a silicon substrate with a 300 nm-thick silicon dioxide layer. Before transferring WS_2 layer on exfoliated graphene flake, oxygen plasma treatment was first introduced to etch the graphene into a desired shape. The WS_2 flake was then aligned to the graphene flake and transferred onto it with the help of a microscope. Au/Cr (50 nm/10 nm) conducting electrodes on top of 2D WS_2/graphene were fabricated using standard electron beam lithography (EBL), metal thermal evaporation, and lift-off processes.

Basic characterization techniques: Raman measurements of the samples were taken using a confocal microscope (WITec, alpha-300) equipped with a 50× objective lens (Zeiss EC Epiplan). The excitation source of the Raman was a 532 nm continuous-wave laser, and the laser beam was focused to the size of about 1 μm on the samples. The electrical properties were measured with a source-meter-unit (Keithley, 2636 B) in a probe station (Lake Shore, CRX-6.5K) in vacuum chamber of 10^{-4} Pa. The extrinsic response measurements were performed using a picosecond pulse laser drive (Taiko PDL M1, PicoQuant) equipped 779 nm laser head with a pulse width of < 500 ps. The generated photocurrent was pre-amplified with a FEMTO HSA-Y-1-60 high-speed current amplifier, after that, it was collected with a lock-in amplifier (UHFLI from Zurich Instruments).

SPCM measurements: scanning photocurrent microscope (SPCM) measurements were performed on a home-built setup. A 780 nm fiber laser (NPI Rainbow 780 OEM) with a pulse width of 80 fs was chopped by a mechanical chopper at 1050 Hz, and then focused onto the sample by a long working distance objective (Olympus LMPLFLN 50×) near the diffraction limit. The generated photocurrent was collected by a lock-in amplifier (Stanford SR830) at the chopped frequency with a background noise of approximately 0.2 pA. The SPCM measurements with the resolution close to the diffraction limit were performed by raster scanning the entire device mounted on a piezoelectric translation stage (Piezoconcept LT3) according to the fixed laser spot.

2PC measurements: For the two-pulse coincidence (2PC) measurements via asynchronous optical sampling (ASOPS), an Optical Sampling Engine (OSE) from Menlo Systems GmbH was used, which features two femtosecond Erbium fiber lasers with a wavelength of λ = 1560 nm, pulse width of ~65 fs and average power of ~90 mW per laser, synchronized with high-accuracy phase-locking electronics. The two pulse trains within one PM1560 (PANDA) fiber are cross-polarized to minimize interference at zero delay. f_{rep} = 100 MHz as the main repetition rate and Δf = 500 Hz as the detuning frequency was used as a trigger signal for a lock-in amplifier. The photodetector channels were connected with 50 Ω matched W-tips and 40 GHz coaxial cables to the UHFLI lock-in amplifier with 1 MΩ input impedance. The 2PC photovoltage was measured using the in-built Periodic Waveform Analyzer (PWA) function of the UHFLI lock-in amplifier, locked at the detuning frequency Δf. Thus, the

periodic signal of the ASOPS sweep with its accumulating delay can be recorded directly, allowing to measure the photovoltage as a function of Δt in real-time. The periodic 2PC curves were recorded with 1024 bins and averaged over up to several giga samples. The PWA operates as a high-speed digitizer synchronized to an oscillator and therefore captures every sample without any dead time while rejecting all non-periodic signal components. The temperature was controlled using a Lake Shore temperature controller (model 336).

Supporting Section 1 : Power density dependent photoresponse in WS_2/graphene heterostructure with 636 nm (above WS_2 bandgap) excitation.

Compared to the hot carrier injected photoresponse by the sub-WS_2 bandgap excitation, here we used a 636 nm laser (above-WS_2 bandgap) to conduct a power dependent photocurrent measurement in WS_2/graphene heterostructure photodetector. With the increase in excitation power, the generated photocurrent exhibits a typical linear to sub-linear transition with the fitted power law of $P \sim 0.92$ (Supporting Figure S1), which reflects a saturated absorption by the WS_2. Besides, the responsivity displays the highest value of approximately 40 A/W with an irradiance of 0.3 mW/cm^2, which quickly decreases due to the saturated absorption.

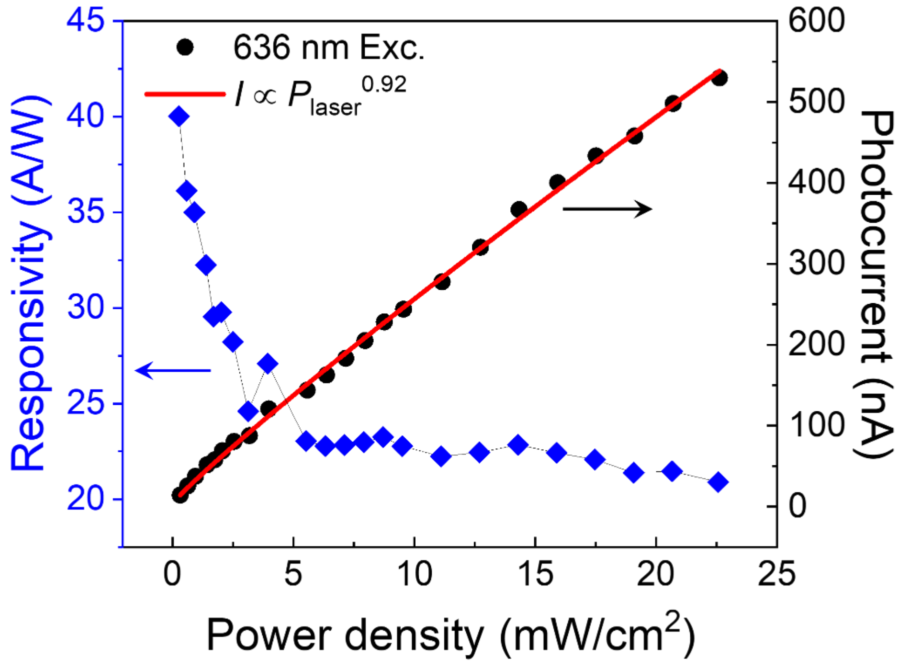

Figure S1. Power density dependence of responsivity (left) and photocurrent (right) under 636 nm (above-WS_2 bandgap) excitation. The red line is a power-law fit with $I_{pc} \approx P^{0.92}$.

Supporting Section 2: Comparison of the power-dependent photocurrent generated in a WS_2/graphene heterostructure between a 779 nm pulsed laser and a 779 nm CW laser

In this section, we present the power-dependent photocurrent generated in a WS_2/graphene heterostructure by a 779 nm continuous wave (CW) laser. As shown in Supporting Figure S2, the CW laser excited photocurrent displays a similar super-linear trend to that from a 779 nm pulsed laser and the fitted power law of $P \sim 1.30$ is a little bit smaller than that from a 779 nm pulsed laser ($P \sim 1.38$). According to the theory for photo-thermionic emission in graphene[1], the generated photocurrent is mostly related to the temperature of hot carriers:

$$PC \propto \Delta T + \frac{\Phi_B}{2k_B T_0^2}\,\Delta T^2 + \cdots,$$

where PC is the generated photocurrent, T_0 is the ambient temperature, ΔT is the increase in electronic temperature, Φ_B is the Schottky barrier between graphene and WS_2, and k_B is Boltzmann's constant. Because thermalized carriers in graphene can reach temperatures significantly higher than the lattice temperature even under CW excitation[2], the power dependence of photocurrent from a pulsed laser and a CW laser are expected to be similar.

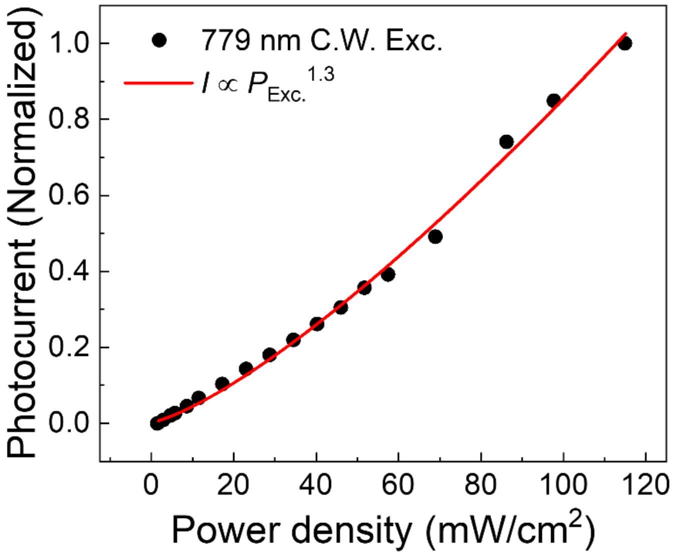

Figure S2., Excitation power density dependence of normalized photocurrent under 779 nm (sub-WS_2 bandgap) CW laser excitation.

Supporting Section 3 : I-V characteristics of a pure graphene photodetector.

We have conducted I-V measurements in pure graphene (Supporting Figure S3), where the on-off ratio of 3 is two and three orders of magnitude smaller than that in our WS_2/graphene heterostructure and WS_2 /graphene/WSe_2 heterostructure, respectively. The dark current is approximately eight orders of magnitude larger than in Figure 1g and 4d.

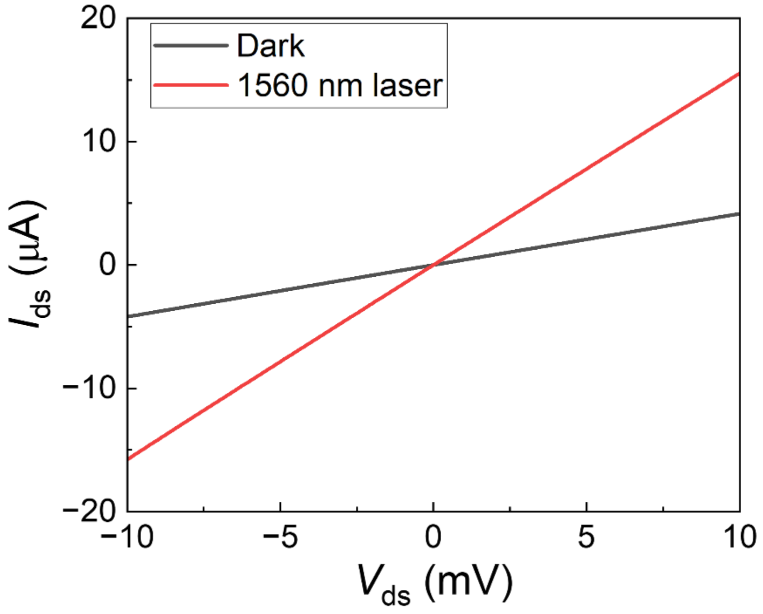

Figure S3. I-V characteristic curves in dark (black) and under 1560 nm illumination (red) for a pure graphene photodetector.

Supporting Section 4 : Exponential fitting of the slow dip component.

We have fitted the slow component of the 2PC measurements in Figure 1i in the main text. For simplicity, we transformed the original data to |ΔPhotocurrent (Normalized)|, which is defined as the difference between the photocurrent (t = 0) and photocurrent (t → ∞). Because the photocurrent intensity for the fast peak component is much higher than that of slow component, we neglected that peak here. The exponential fitting yields a response time for the slow component of approximately 468.8 ps.

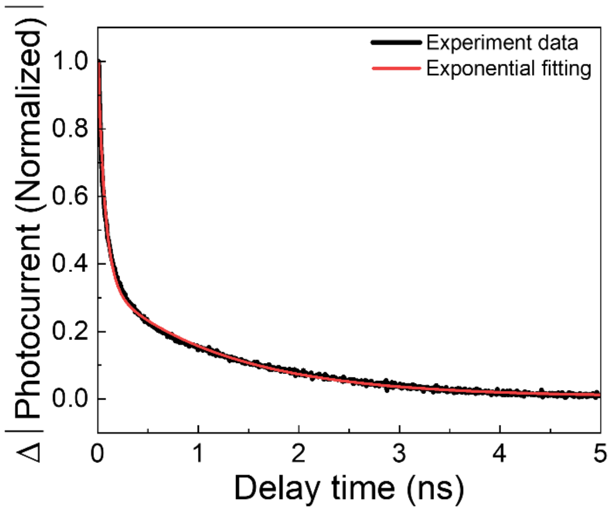

Figure S4. Exponential fitting of the slow dip component, where the intrinsic response time is fitted as 468.8 ps. |ΔPhotocurrent (Normalized)| is defined as the difference between the photocurrent (t = 0) and photocurrent (t → ∞).

Supporting Section 5 : Power dependence of 2PC measurements in WS_2/graphene.

We have conducted power-dependent 2C measurements in WS_2/graphene photodetector by inserting fiber attenuators (3 dB and 10 dB). With the decrease in laser power, the fast peak component exhibits a slightly extended lifetime (Supporting Figure S5), presumably due to the effects of increased photo-resistance[3] and enhanced exciton-exciton annihilation[4].

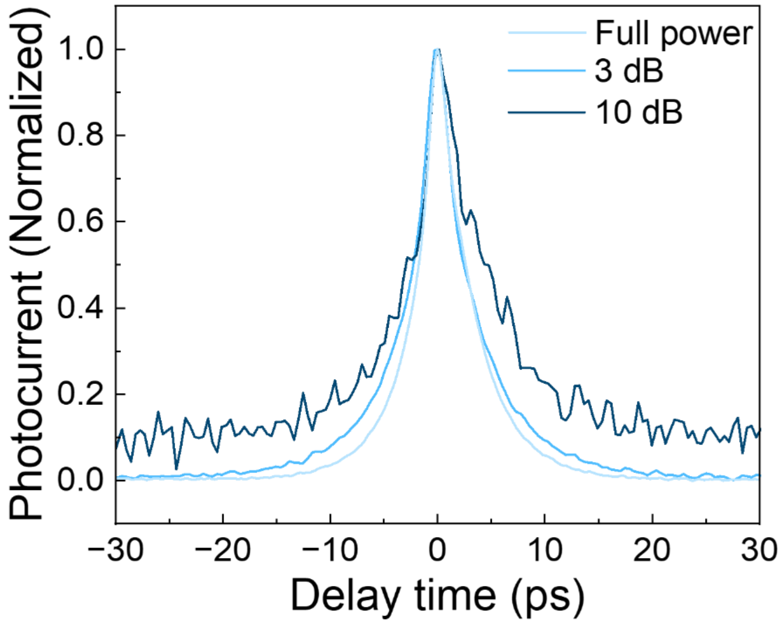

Figure S5. Power dependence of 2PC measurement in WS_2/graphene. The excitation power is 90 mW, 45 mW and 9 mW for full power, 3-dB and 10-dB, respectively.

Supporting Section 6 : Reproducibility of the intrinsic response signal.

The observed intrinsic response signal in Figure 2 in the main text was reproduced in another device. Supporting Figure S5 a shows the optical image of the device, where electrodes 1 and 2 were fabricated on top of the WS_2 layer and electrode 3 was on the bottom of the WSe_2 layer. During the 2PC measurements, only electrodes 1 and 2 were connected and hence the device configuration was similar to that of Figure 1 a in the main text. In 2PC measurements, the device exhibits similar photocurrent lifetimes with two different components, where the fast peak component was independent of the external bias and the slow dip component disappeared under sufficient external bias (Supporting Figure S6 b-c). In addition, with the decrease in temperature, the fast peak component increased from 1.7 ps at room temperature to 3.1 ps at 8 K (Supporting Figure S6 d-e). All the above phenomena are consistent with the observations in the WS_2/graphene device in the main text.

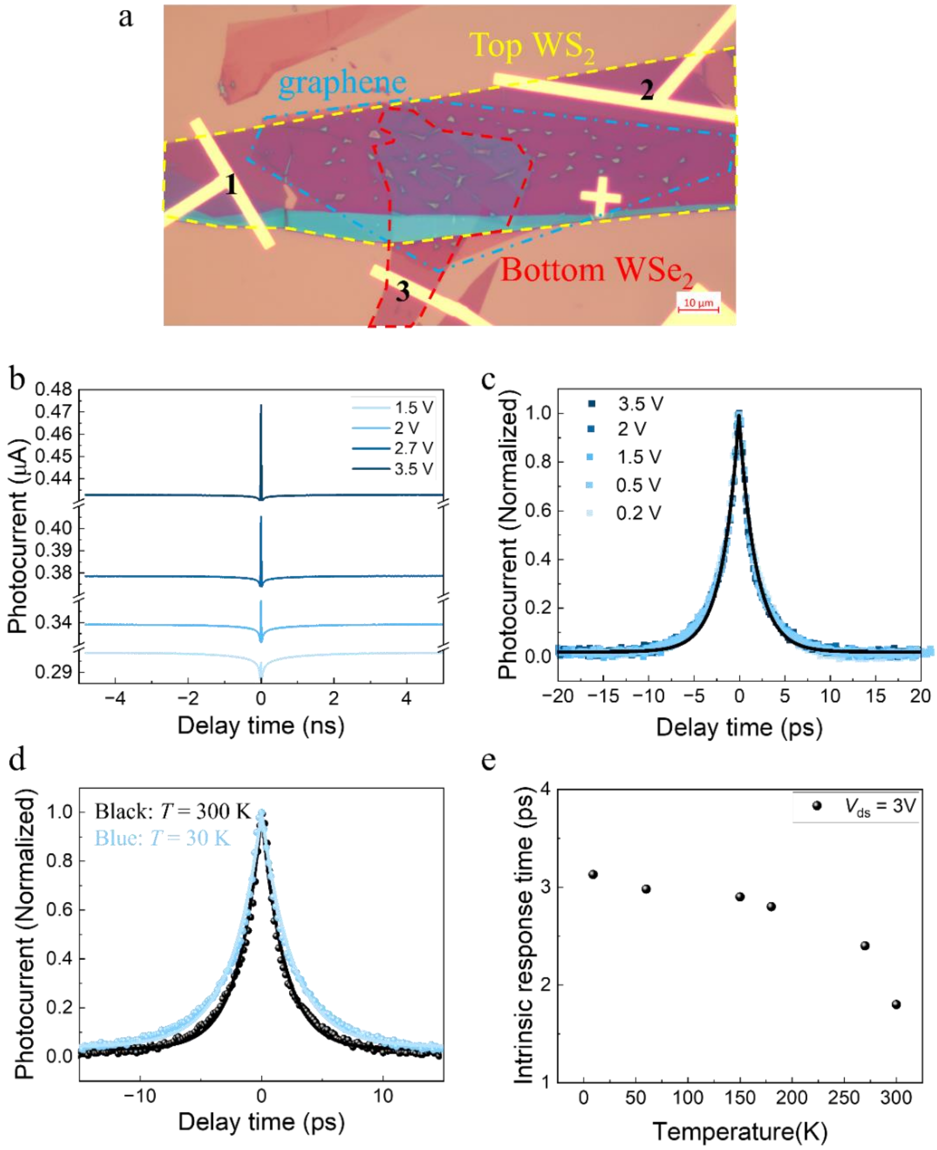

Figure S6. 2PC measurement of a second device. (a) Optical image of the WS_2/graphene/WSe_2 photodetector, where electrodes 1 and 2 are on top of the WS_2 layer and electrode 3 is on bottom WSe_2 layer. During the 2PC measurements, only electrodes 1 and 2 were connected and hence the device configuration was similar to that of Figure 1 a in the main text. (b) External voltage dependence of the 2PC results, where the slow component decreases with the increase in external bias. (c) Bias dependence of the 2PC results for delay times from -30 ps to 30 ps. (d) Temperature dependence of the 2PC results for time delay from -30 ps to 30 ps. (e) Temperature dependence of the intrinsic response time originating from fast component.

Supporting Section 7 : Calculation of the photoresponsivity for WS_2/graphene at 1560 nm laser and 779 nm impulse laser.

The photoresponsivity of the WS_2/graphene photodetector (Figure 1a in the main text) towards 1560 nm laser has been calculated according to the formula:

$$R = \frac{I_{\text{photocurrent}}}{P_{\text{input}}}.$$

$I_{\text{photocurrent}}$ is the generated photocurrent by the 1560 nm laser. Here, we use the value of 0.51 μA measured under an external bias of 3 V (data from Figure 2a in the main text). P_{input} is the input laser power on the active region of the device, which can be expressed as:

$$P_{\text{input}} = P_{\text{laser}} \times \frac{A_{\text{device}}}{A_{\text{illumination}}},$$

where P_{laser} is the measured laser power and A_{device} as well as $A_{\text{illumination}}$ are the device and illumination area, respectively. In our measurements, we used a laser fiber to introduce the light. In the ASOPS setup, the output laser power of laser A+B is 180 mW while the measured laser power out from the laser fiber is 22.5 mW. In addition, for our WS_2/graphene heterostructure photodetector (Figure 1a in the main text), the active area is 100 μm^2. The diameter of laser spot was roughly measured as 0.12 cm and, thus, the illumination area is approximately 0.0113 cm^2. In this case, the P_{input} can be calculated as:

$$P_{\text{input}} = P_{\text{laser}} \times \frac{A_{\text{device}}}{A_{\text{illumination}}} = 22.5 \text{ mW} \times \frac{100 \ \mu\text{m}^2}{0.0113 \text{ cm}^2} = 1.99 \ \mu\text{W}.$$

In this case, the photoresponsivity is $R = \frac{I_{\text{photocurrent}}}{P_{\text{input}}} = \frac{0.51 \ \mu\text{A}}{1.99 \ \mu\text{W}} \approx 0.26$ A/W.

Similarly, the responsivity of WS_2/graphene towards a 779 nm impulse laser (Figure 1e in the main text) is calculated in the same way. Here, P_{laser} was varied from 2.5 μW to 177.5 μW. The measured diameter of laser spot was 0.1 cm, slightly smaller than that of 1560 nm laser. In this case, the maximum (minimum) P_{input} is calculated as:

$$P_{input} = P_{laser} \times \frac{A_{device}}{A_{illumination}} = 177.5\ \mu W\ (2.5\ \mu W) \times \frac{100\ \mu m^2}{0.00785\ cm^2} \approx 25\ nW\ (0.352\ nW).$$

The maximum (minimum) photoresponsivity is $R = \frac{I_{photocurrent}}{P_{input}} = \frac{225\ nA}{25\ nW} \approx 9\ A/W$ ($\frac{1.25\ nA}{0.352\ nW} \approx 3.55\ A/W$), which follows the trend in Figure 2e in the main text.

Supporting Section 8 Comparison of the photocurrent intensity in devices with and without the slow component.

We have compared the photocurrent intensity in two different types of devices with and without a slow component in the 2PC signal results under the same measurement conditions. The one without the slow component (Supporting Figure S7b) demonstrates one order of magnitude lower photocurrent (Supporting Figure S7d) than that of the device (Supporting Figure S7a) with the slow component (Supporting Figure S7c). The results are consistent with our hypothesis that the slow dip component orignates from trapped holes and contributes to a long circulation of the injected electrons.

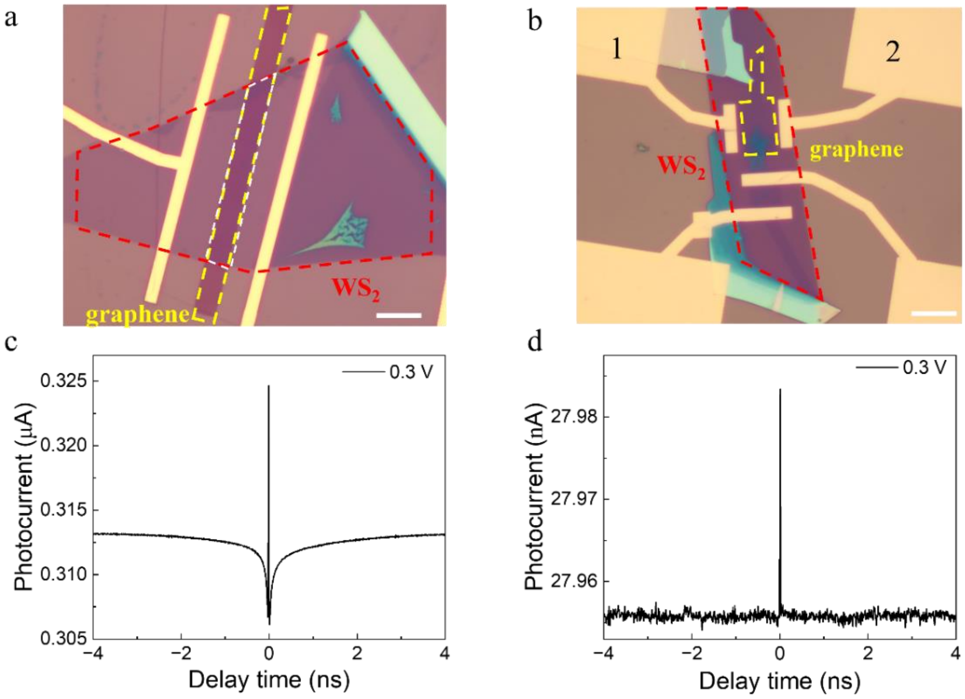

Figure S7. (a) and (b) Optical images of WS_2/graphene heterostructures. The scale bars are 10 μm. (c) and (d) Corresponding 2PC measurements for the devices in a and b, respectively.

Supporting Section 9 Fabrication procedure of WS_2/graphene photodetector arrays.

To demonstrate the superiority of our WS_2/graphene heterostructure for fast photodetection, a WS_2/graphene photodetector array was fabricated as shown in Supporting Figure S7. In detail, a thin layered graphene flake with a size around 60 μm × 100 μm was firstly exfoliated on PDMS (Supporting Figure S8a) and transferred onto SiO_2/Si substrate. Then an oxygen plasma treatment was used to etch the graphene to form a periodic graphene pattern (Supporting Figure S8b). After that, a thin layered WS_2 flake with a uniform thickness was transferred onto the graphene array to form a WS_2/graphene heterostructure array (Supporting Figure S8c). Finally, electrodes were defined onto the heterostructure array via standard electron beam lithography, thermal evaporation and lift-off processes (Supporting Figure S8d).

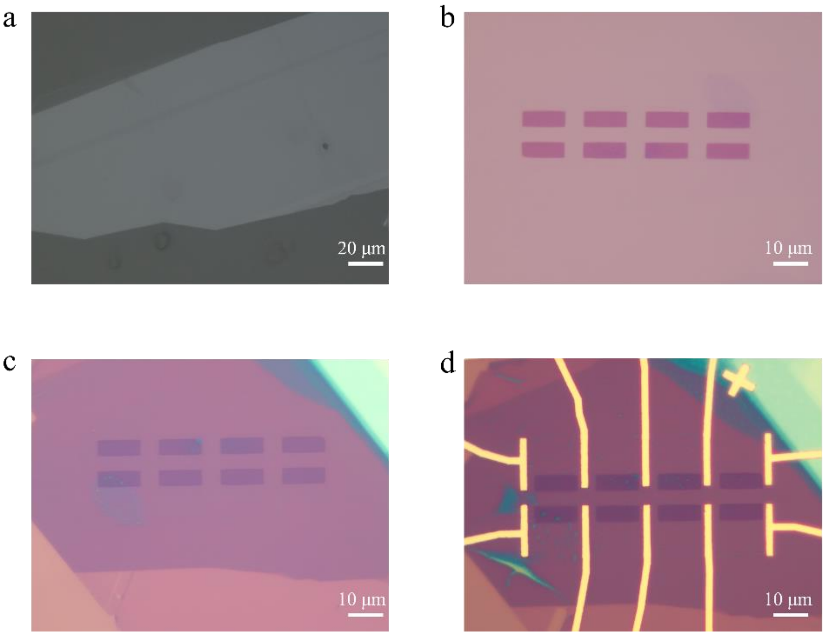

Figure S8. (a) Exfoliated graphene on PDMS. (b) Plasma etched graphene array on SiO_2/Si substrate. (c) WS_2/graphene heterostructure array on SiO_2/Si substrate. (d) WS_2/graphene photodetector array.

Supporting Section 10 : 2PC measurements in WSe_2/graphene/WS_2 veritical heterostructure.

The supplemantary 2PC measurement results of WSe_2/graphene/WS_2 veritical heterostructure is provided in Supporting Figure S9, where a similar phenomena to that in WS_2/graphene in the main text has been observed.

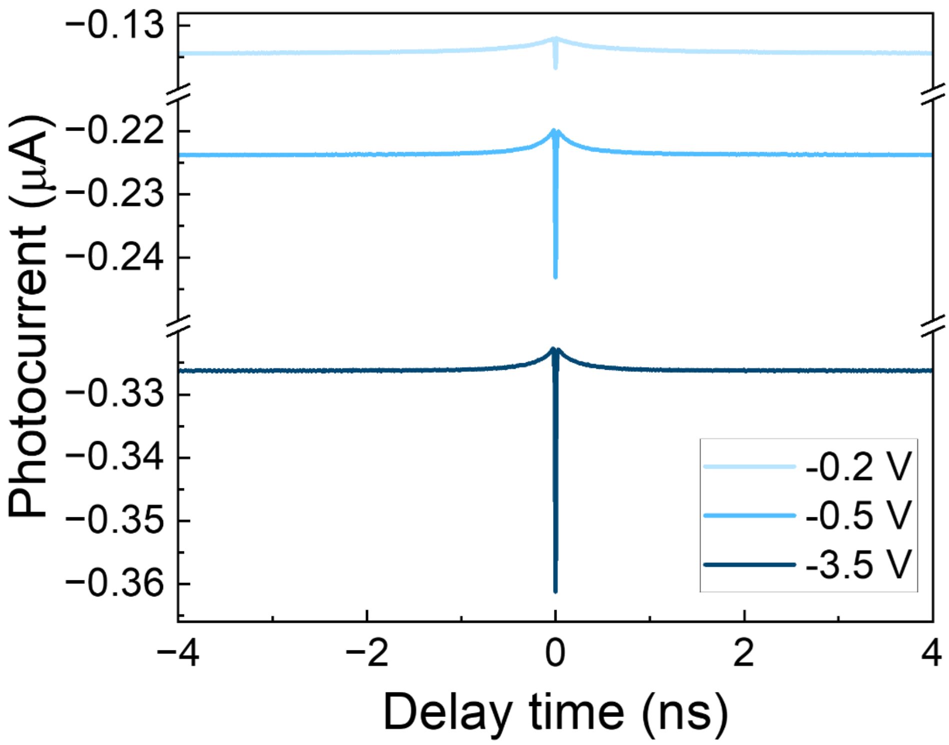

Figure S9. Bias dependence of the 2PC results in WSe_2/graphene/WS_2.

Supporting Section 11 : Supporting results for the laser repetition related extrinsic response time and 3-dB bandwidth in WSe_2/graphene/WS_2 photodetectors.

The supporting extrinsic photoresponse time of the WSe_2/graphene/WS_2 device towards 779 nm illumination with different laser repetition frequencies are presented in the Supporting Figure S10. The red lines indicate 90% to 10% of the photocurrent decay.

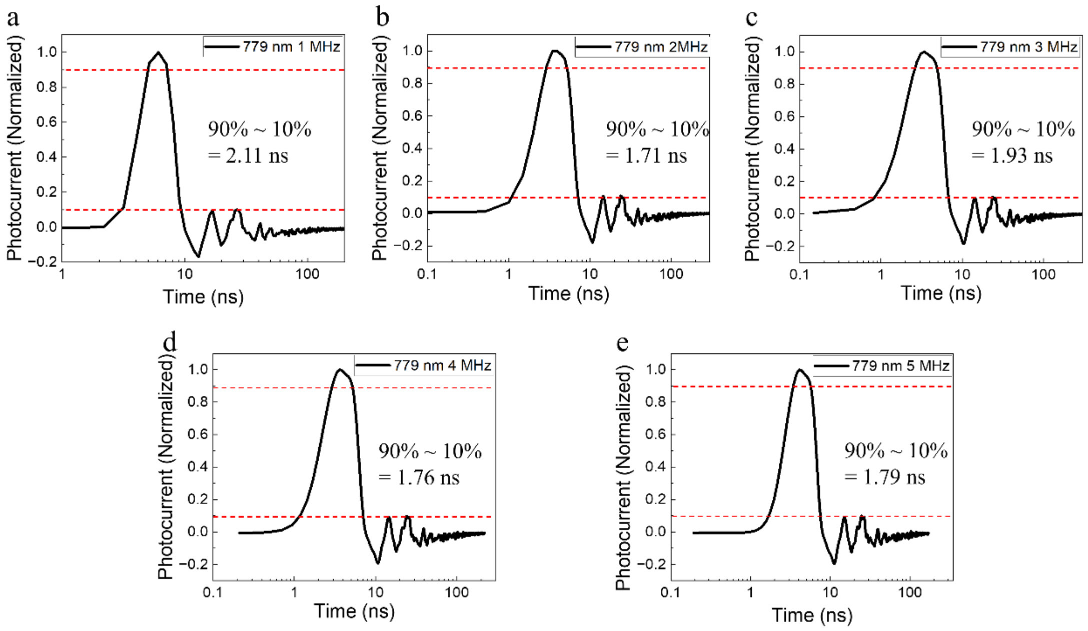

Figure S10. Extrinsic response time measured in WSe_2/graphene/WS_2 phtodetector with 779 nm impulse laser in semi-log scale. (a-e) Extrinsic response time measured in WSe_2/graphene/WS_2 photodetector with 779 nm impulse laser in semi-log scale. The input laser repetition frequencies were changed from 1 MHz to 5 MHz. All data were obtained at 0 V external voltage.

The supplementary 3-dB bandwidth of the WSe_2/graphene/WS_2 device towards 779 nm illumination with different laser repetition frequencies are presented in Supporting Figure. S11. The red lines indicate 3-dB bandwidth.

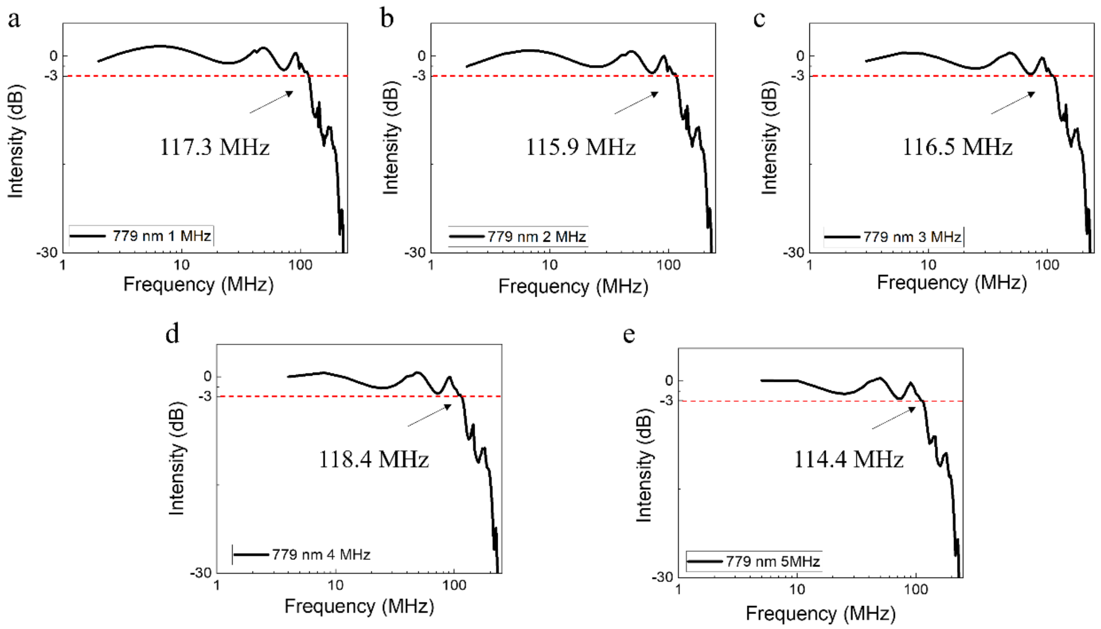

Figure S11. Calculated 3-dB bandwidth for WSe_2/graphene/WS_2 photodetector with 779 nm impulse laser. (a-e) The input laser repetition frequencies are 1 MHz to 5 MHz for the results.

Supporting Section 12 : Hot carrier dynamics in WS_2/graphene/WSe_2 tri-layer heterostructures.

In this section, we propose a possible explanation for the hot carrier dynamics in the WS_2/graphene/WSe_2 tri-layer heterostructure (Supporting Figure S12). After excitation by the 1560 nm laser, the generated hot electrons in graphene will be rapidly injected into WS_2 and cool in several picoseconds, which is similar to the dynamics in the WS_2/graphene lateral device. According to the band alignment of the tri-layer heterostructure[5], the generated holes should be transferred into the WSe_2 layer due to its higher valence band edge. Here, we argue that trap states also exist on the surface of WSe_2. In this case, because our tri-layer heterostructure is not an ideal vertical structure, where the gold electrodes were not defined exactly on the top and bottom of the tri-layer overlap, the transferred holes from graphene to WSe_2 will not be immediately collected by the electrodes but are trapped by the surface defects, which presumably introduces the slow component.

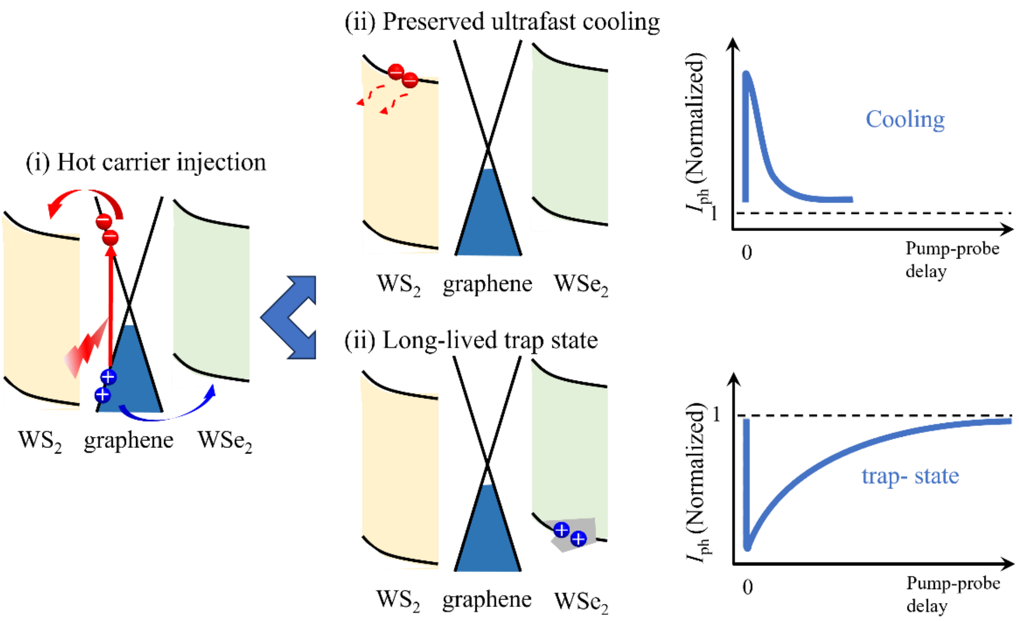

Figure S12. Schematic illustration of hot carrier dynamics in WS_2/graphene/WSe_2.

Supporting Section 13 : Temperature-dependent 2PC photoresponse in a WS_2/graphene/WSe_2 heterostructure.

The temperature-dependent 2PC photoresponse of a WS_2/graphene/WSe_2 heterostructure is presented in Supporting Figure S13. The extended intrinsic response time varies from 2.5 ps at room temperature to 3.87 ps at 80 K (Figure S13b). This decrease in temperature is similar to the phenomenon in Figure 2c-d in the main text, which suggests the same hot carrier cooling dynamics as that in WS_2/graphene.

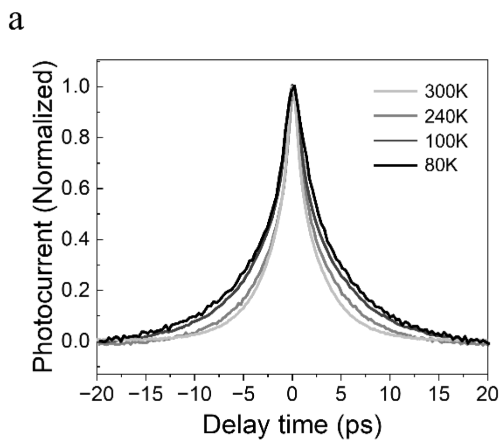

Figure S13. (a) Temperature dependence of 2PC results in tri-layer WS_2/graphene/WSe_2 heterostructure for a time delay from -20 ps to 20 ps. (b) Corresponding temperature-dependent intrinsic response times.

Supporting Section 14 : Extrinsic response time of WS_2/graphene/WSe_2 devices towards a 1310 nm square pulse laser.

For a better demonstration of the superiority of our WS_2/graphene/WSe_2 vertical devices with ultrafast hot carrier cooling and built-in electric field, the extrinsic response time closer to telecom wavelength has been measured via a 1310 nm square pulse laser. As shown in the Supporting Figure S14, the photocurrent rise (a) and decay (b) times are approximately 60 ns, which is slightly slower than the results obtained with a 779 nm impulse laser. We attribute this phenomenon to the weaker peak power of the 1310 nm square pulse laser compared to the 779 nm impulse laser, which induces a lower photoconductivity of WS_2 and WSe_2 and enhance the RC time of the devices. It should be noted that this result is still better than most photodetectors operating at telecom wavelengths including the commercial ones, for example, Type II InAs/GaSb photodetectors (~100 ns) and HgCdTe photodetectors (~500 ns)[4,5].

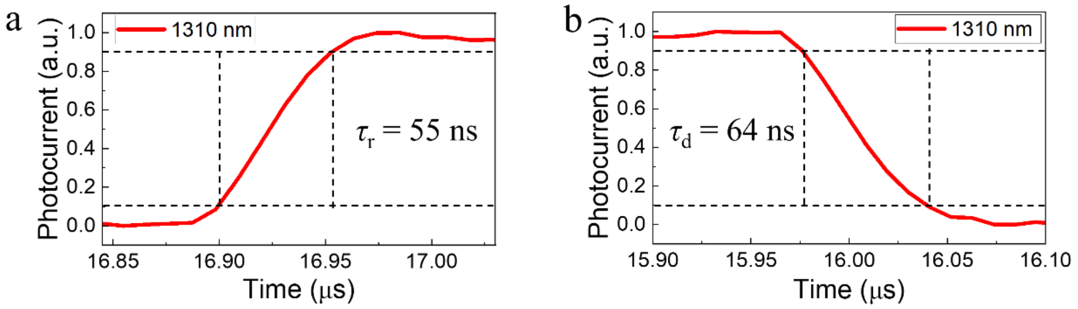

Figure S14. Photocurrent rise (a) and decay (b) time towards 1310 nm (sub-WS_2 bandgap) square pulse laser.

Supporting References:

(1) Massicotte, M.; Schmidt, P.; Vialla, F.; Watanabe, K.; Taniguchi, T.; Tielrooij, K. J.; Koppens, F. H. L. Photo-thermionic effect in vertical graphene heterostructures. *Nat. Commun.* **2016**, *7*, 12174.

(2) Gabor, N. M.; Song, J. C. W.; Ma, Q.; Nair, N. L.; Taychatanapat, T.; Watanabe, K.; Taniguchi, T.; Levitov, L. S.; Jarillo-Herrero, P. Hot Carrier-Assisted Intrinsic Photoresponse in Graphene. *Science* **2011**, *334* (6056), 648-652.

(3) Sun, D.; Aivazian, G.; Jones, A. M.; Ross, J. S.; Yao, W.; Cobden, D.; Xu, X. D. Ultrafast hot-carrier-dominated photocurrent in graphene. *Nat. Nanotechnol.* **2012**, *7* (2), 114-118.

(4) Yuan, L.; Huang, L. B. Exciton dynamics and annihilation in WS_2 2D semiconductors. *Nanoscale* **2015**, *7* (16), 7402-7408.

(5) Long, M. S.; Liu, E. F.; Wang, P.; Gao, A. Y.; Xia, H.; Luo, W.; Wang, B. G.; Zeng, J. W.; Fu, Y. J.; Xu, K.; et al. Broadband Photovoltaic Detectors Based on an Atomically Thin Heterostructure. *Nano Lett.* **2016**, *16* (4), 2254-2259.

(6) Rogalski, A. Infrared detectors: status and trends. *Prog. Quantum Electron.* **2003**, *27* (2), 59-210.

(7) Rogalski, A. Progress in focal plane array technologies. *Prog. Quantum Electron.* **2012**, *36* (2), 342-473.
